# Desensitization Protocol for Cemiplimab-Related Infusion Reaction in Cutaneous Squamous Cell Carcinoma: A Case Report and Literature Review

**DOI:** 10.3390/curroncol30070491

**Published:** 2023-07-14

**Authors:** Marco Banini, Viola Salvestrini, Alessandra Vultaggio, Margherita Perlato, Valentina Mecheri, Cecilia Cerbai, Vieri Scotti, Andrea Matucci, Monica Mangoni, Lorenzo Livi, Pierluigi Bonomo

**Affiliations:** 1 Department of Biomedical, Experimental, and Clinical Sciences “Mario Serio”, University of Florence, L.go Brambilla 3, 50134 Florence, Italy; 2 Immunoallergology Unit, Careggi University Hospital, 50134 Florence, Italy; 3 Department of Radiation Oncology, Azienda Ospedaliero-Universitaria Careggi, 50134 Florence, Italy

**Keywords:** cutaneous squamous cell carcinoma, Cemiplimab, drug-related infusion reaction, drug desensitization

## Abstract

Background: The landscape of systemic therapies for advanced non-melanoma skin cancers has been revolutionized by the advent of immunotherapy. Cemiplimab is the only immune checkpoint inhibitor (ICI) approved by the European Medicine Agency for recurrent/metastatic cutaneous squamous cell carcinoma (cSCC). Its excellent efficacy outcomes are achieved due to its good tolerability profile. The drug-related hypersensitivity reaction (HSR) is a well-known issue in oncology, but it is rarely reported in respect to immune checkpoint inhibitors. Cemiplimab is among the agents with the best infusion tolerability profiles. Clinical practice guidelines in this field are lacking. Results: We report on the successful management of a severe infusion reaction induced by Cemiplimab in a patient with cSCC based on a desensitization protocol, which led to adequate treatment delivery and prolonged clinical benefit. A review of the available literature on HSR rates and its management with ICIs, and on drug desensitization (DD) protocols and their efficacy, was conducted to highlight the limited knowledge on this topic and its importance. Conclusion: Our experience highlights the need for a DD protocol in order to improve the treatment of HSRs, particularly when elicited by an immunotherapy agent, preventing treatment discontinuation and preserving its efficacy.

## 1. Introduction

Cutaneous squamous cell carcinoma (cSCC) is the second most common skin cancer and has undergone a clear rising trend over the past 10 years, now comprising about 20% of all non-melanoma skin cancers [1], with more than 90% of cases treatable with surgery or radiotherapy [2]. However, until recently, no standard first-line regimen had been defined for recurrent/metastatic (R/M) cSCC [3]. The landscape of systemic therapies for advanced non-melanoma skin cancers has been revolutionized by the advent of immune checkpoint inhibitors (ICIs) [4]. Cemiplimab is a human monoclonal antibody that binds to programmed death receptor-1 (PD-1) and blocks its interaction with programmed death ligands 1 (PD-L1) and 2 (PD-L2). Currently, it is the only immune checkpoint inhibitor approved by the European Medicine Agency for R/M cSCC [5], based on the results of the pivotal open-label, non-randomized, multi-center EMPOWER-CSCC-1 phase I–II trial. A total of 219 subjects with R/M cSCC not amenable to surgery or radiation were included in this study. Excellent efficacy outcomes were achieved with a good tolerability profile, whereby grade 3–4 treatment-related adverse events (TRAEs) were extremely rare, with the most common being pneumonia (2.3%), hepatitis (1.8%) and diarrhea (1%), and only 8% of patients had to discontinue treatment due to an AE [6,7,8].

More recently, the efficacy results demonstrated in a first-line setting also led to the testing of Cemiplimab in a neoadjuvant setting for resectable stage II–IV (M0) cSCC. Interestingly, about 50% of patients experienced a pathological complete response with four neoadjuvant infusions, confirming the huge impact that this drug is having on the natural history of the disease [9].

The hypersensitivity reaction (HSR) induced by drug infusion is a well-known issue in oncology, especially when considering cytotoxic agents such as taxanes and platinum compounds. The HSR incidence rate reported for taxanes is 30%, which is reduced to 2–4% with appropriate premedication and 10–27% for platinum compounds [10]. Occasionally such reactions can be severe or life-threatening and may result in drug suspension or interruption, compromising patients’ therapeutic pathways. Overall, IRs are rarely reported in respect to immune checkpoint inhibitors (ICIs). According to the literature, Cemiplimab is among the agents with the best infusion tolerability profiles; in the pivotal EMPOWER-CSCC-1 trial, HSRs were reported in less than 4% of patients, with no grade 3–4 reactions [6]. Consequently, clinical practice guidelines in this field are lacking. Here, we report on the successful management of a severe infusion reaction induced by Cemiplimab in a patient with cSCC based on a desensitization protocol, which led to adequate treatment delivery and prolonged clinical benefit.

## 2. Detailed Case Description

An 82-year-old man with a previous history of squamous cell carcinoma of the larynx presented with a recurrent cSCC in the right pretragal area.

The patient was a former heavy smoker (approximately 50 pack/year), with a diagnosis of diabetes mellitus type 2, arterial hypertension and inguinal hernia in his medical history. In 2012 he was diagnosed with squamous cell carcinoma of the right vocal cord, which was managed via endoscopic resection of 2/3 of the anterior parts of right false vocal cord and adjuvant radiotherapy targeting the tumor bed with prophylactic neck irradiation. In April 2019, the patient underwent surgical resection of a scalp lesion located in the fronto-parietal region, histologically confirmed as a second primary cSCC, stage pT3. In July 2019, he underwent a right parotidectomy and ipsilateral neck lymph node dissection (levels IB–IV), which confirmed the presence of recurrent squamous cell carcinoma in the parotid without pathological involvement of the removed neck lymph nodes. In October 2019, head and neck contrast-enhanced computed tomography (CE-CT) showed a recurrent lesion in the right external auditory canal. An excisional biopsy of the right ear lesion was performed, with histological confirmation of invasive cSCC. In December 2019, external beam radiation therapy with palliative intent was prescribed, for a total delivered dose of 36 Gy in 18 fractions.

**Figure 1:**
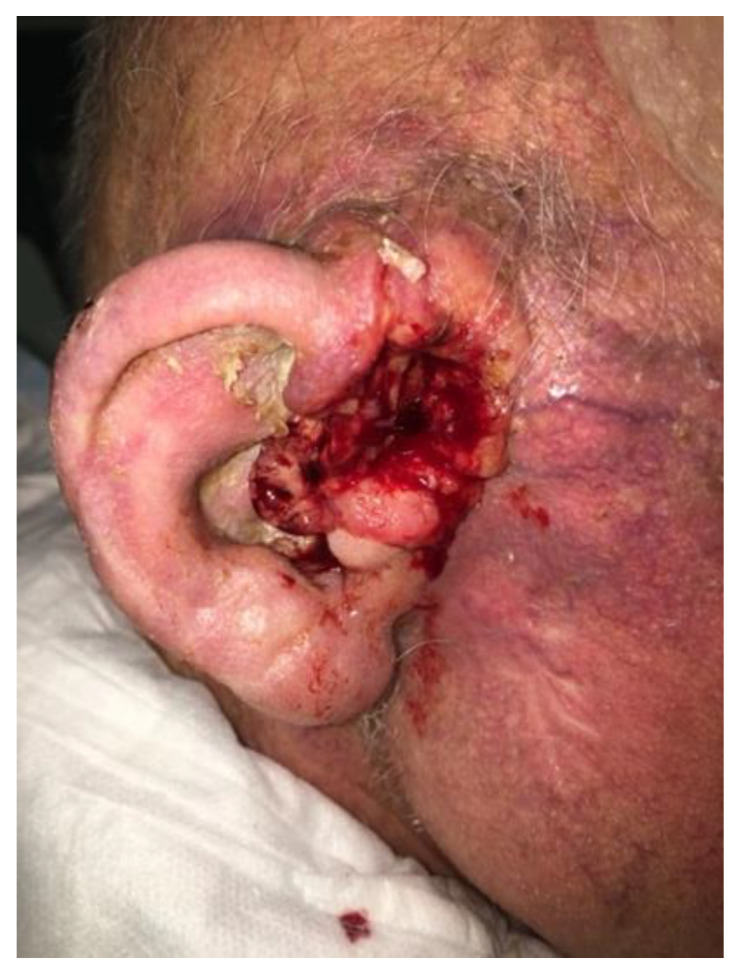
Clinical disease status before start of Cemiplimab.

In February 2020, the patient presented for clinical re-evaluation with a 3 × 4 cm mass in the pretragal area, with skin ulceration and easy bleeding, and with the surrounding ear’s auricle presenting as erythematous and swollen, based on which we suspected tumoral invasion. A CE-CT scan confirmed the presence of pathological tissue extending to the right parotid bed, obliterating the external auditory canal and infiltrating the ear’s auricle (Figure 1).

Thus, the patient started immunotherapy with Cemiplimab 350 mg administered intravenously (IV), scheduled every three weeks; the drug infusion did not include any premedication as per local protocol. The first infusion was well tolerated, whereas ten minutes after the beginning of the second administration, the patient experienced a severe HSR, presenting with G3 (CTCAE v 5.0) bilateral lumbar pain, sudden G2 erythema and G3 peripheral oxygen desaturation, which decreased to 85% in room air. The patient did not develop either hypotension or fever. He had a complete regression of symptoms after infusion interruption, the administration of half a vial of 1000 mg hydrocortisone intravenously (IV) and inhalation oxygen therapy at 2 L/min. Half an hour later, there was a complete regression of respiratory symptoms, with no need of further inhalation oxygen therapy and a recovery of oxygen saturation to 94% in room air. For the allergy work-up, he was referred to the Immuno-allergology Unit of our University Hospital, where the reaction was defined as Grade 3 according to Brown’s Classification [11]. The skin testing result was negative. Specifically, a full-strength prick test (1.4 mg/mL) and intradermal tests with Cemiplimab at concentrations of 1:100 (0.014 mg/mL), 1:10 (0.14 mg/mL) and 1:1 (1.4 mg/mL) were performed, with histamine as a positive control, +++. Considering the severity of the reaction, and the absence of any alternative therapeutic approach for the patient, a 16-step drug desensitization (DD) protocol was developed for a target dose of 350 mg of Cemiplimab. Premedication with H1 and H2 blockers and systemic corticosteroids was included. The desensitization solutions and steps followed are described in Table 1.

The patient was monitored in a day hospital setting with close supervision by an allergist and a nurse. His vital signs were monitored every 15 min and medications for emergency use were kept at his bedside. Desensitization was completed in approximately 7 h and 7 min. The patient was monitored for any HSRs for 1 h following each DD.

**Figure 2:**
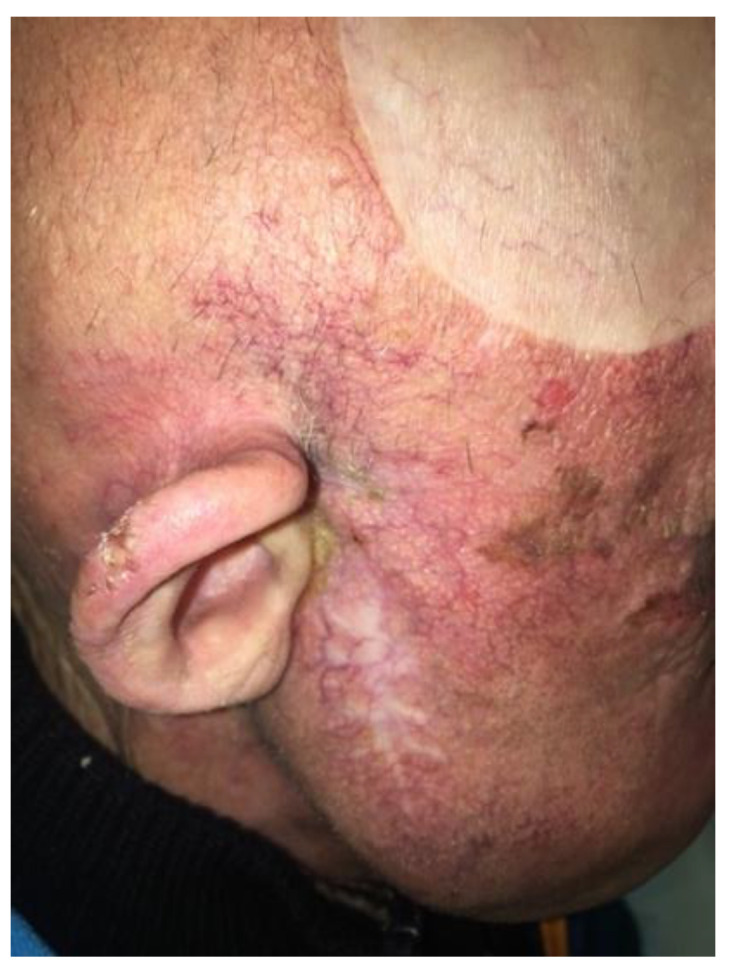
Clinical response after 3 months of treatment with Cemiplimab, which was conducted, from the third cycle, through our immunologists’ desensitization protocol.

In May 2020, after two DD procedures, the patient underwent a medical evaluation, presenting with complete clinical disease remission (Figure 2).

Up to May 2021, the patient received 20 DDs in total with no breakthrough reactions. Treatment was then interrupted due to the diagnosis of another metachronous squamous cell carcinoma of the oral cavity located in the right retromolar trigone area, infiltrating the right mandibular branch, confirmed histologically and radiologically. The response to immunotherapy was maintained in the cutaneous ear and parotid area, with no local signs of treatment-related toxicity. The patient then started second-line weekly paclitaxel, which was interrupted in May 2022 due to intolerable neurotoxicity. At the last follow-up on 26th of January 2023, the responses for both tumors were confirmed and maintained.

## 3. Discussion

In this case study, we report the management of a severe immediate HSR to Cemiplimab by performing a DD protocol. To our knowledge, this is the first successful DD protocol planned for Cemiplimab and published in the literature that led to adequate treatment delivery and prolonged clinical benefit.

HSRs resulting from the use of ICIs in non-melanoma skin cancers are rarely reported. Most of these reactions are mild to moderate with symptoms such as skin rash, pruritus, headache, fever and nausea. Severe infusion events are rare but may be fatal without appropriate management. There are few data available regarding HSRs and their management during the infusion of ICIs. Hence, we conducted a scoping review of the literature to obtain the data available on non-melanoma skin cancers. We evaluated phase I–II trials published in the last four years reporting toxicity data and treatment-related HSR rates associated with ICIs in non-melanoma skin cancer patients. To our knowledge phase III trials or large real-world datasets are still missing in the literature. We excluded case reports and small retrospective studies involving fewer than 50 patients. The studies included in our review and their relevant results are summarized in Table 2.

In 2018, Midgen et al. reported the safety data from the expanding cohort of a phase I/II study testing Cemiplimab on 85 patients affected by metastatic cSCC. Cemiplimab was administered IV at a dose of 3 mg/Kg every 2 weeks, and only three treatment-related infusion reactions (3.5% of total TRAEs), of Grades 1–2, were reported [6]. In 2020 were also reported the safety results from a cohort of 78 locally advanced cSCC patients, confirming the same overall toxicity profile (14.1% TRAEs G ≥ 3), with HSRs not being specifically reported, not having registered any Grade ≥ 3 or any Grade ≥ 10% [7]. Rischin et al. reported, in 2020, the safety results from the fixed dose cohort of the same trial, where 56 patients affected by metastatic cSCC were treated with Cemiplimab administered IV at a fixed dose of 350 mg every three weeks, which is the actual approved regimen. The rate of HSR during infusion was again not reported, not occurring in ≥5% of the patient population or in those with Grade ≥ 3 [8]. Considering other ICIs, Grob et al. in 2020 tested safety and efficacy of Pembrolizumab in 109 R/M cSCC patients in the Keynote-629 trial. Patients were treated with Pembrolizumab, administered IV at a fixed dose of 200 mg every 3 weeks. Only 1 HSR was reported, being Grade 3 [12]. In the same year, Maubec et al. reported the data of 55 patients with unresectable cSCC treated with 200 mg first-line Pembrolizumab every 3 weeks in the CARSKIN trial, where no HSRs occurred [13]. With respect to Merkel cell carcinoma (MCC), Nghiem et al. reported data from the Keynote-017 trial. Fifty patients with advanced MCC were enrolled and treated with Pembrolizumab administered IV at a dose of 2 mg/Kg every 3 weeks. Only 1 infusion-related reaction was reported [14]. In the aforementioned studies, the authors never reported or suggested a specific method of management for such HSRs. D’angelo et al. documented the updated toxicity profile from the Javelin 200 Merkel trial. Patients with stage IV MCC were treated with second-line Avelumab administered IV at a dose of 10 mg/Kg every 2 weeks in 1 h infusions. Infusion reactions were reported in 21.3% patients, and were all Grade 1–2 [15]. Due to the high incidence of infusion-related reactions, the protocol was amended and included premedication with an antihistamine (e.g., diphenhydramine) and acetaminophen 30–60 min before at least the first four infusions [18]. In 2018, D’Angelo et al. also reported the safety of 39 patients with stage IV MCC treated with Avelumab as a first-line treatment. Notably, 23.1% patients experienced infusion-related HSRs, with one reaction being Grade 3 and leading to the discontinuation of treatment for two patients. Similarly, patients were treated with premedication as per the protocol amendment [16]. Lastly, in respect to basal cell carcinoma (BCC), Stratigos et al. highlighted the results of Cemiplimab in patients with locally advanced disease after the failure of Hedgehog inhibitor therapy. The toxicity profiles of Cemiplimab administered IV at fixed dose of 350 mg every 3 weeks were reported for 84 patients treated in a phase II trial. The HSR rate was not reported, given that it did not reach the 10% threshold with any grade and there were not any reactions of Grade 3 or above [17]. In summary, with the exception of Avelumab in MCC, the infusion-related reaction rates for ICIs in non-melanoma skin cancers are very rarely reported, and therefore, there are no specific indications for their management. Within most protocol trials and product labels, the following general recommendations are reported: for Grade 1 symptoms, restart infusion at 50% speed, with the administration of diphenhydramine 50 mg (or equivalent) and/or acetaminophen/paracetamol 325 mg to 1000 mg at least 30 min prior to subsequent Cemiplimab infusions. For Grade 2 symptoms, stop infusion and start symptomatic therapy; this may restart at 50% speed when symptoms resolve. The administration of medications such as diphenhydramine 50 mg (or equivalent) and/or acetaminophen/paracetamol 325 mg to 1000 mg is recommended at least 30 min prior to subsequent Cemiplimab infusions. Corticosteroids (up to 25 mg of hydrocortisone or equivalent) may be used. For reactions of Grade 3 or above, where symptoms do not recover rapidly after infusion suspension and symptomatic treatment, more intensive support therapy is required, and permanent discontinuation is recommended.

### Drug Desensitization Protocols

Desensitization is a process designed to safely reintroduce a drug to a patient who had developed an HSR to it. It is especially indicated with respect to reactions mediated by masts cell activation to the first-line drug therapy, without comparable alternatives [19]. It is largely applied to platins and monoclonal antibodies, but has rarely been applied to ICIs [20]. DD consists of incremental escalation of the sub-optimal doses of the culprit drug until the required dose is reached, and it induces temporary tolerance, which protects the patient from anaphylaxis, taking advantage of the inhibitory mechanisms that prevent activated mast cell signal transduction and pro-inflammatory mediator release [21]. Desensitization protocols may vary between different institutions as they may also need to be designed for a single patient and the specific drug involved. The critical aspect to be pointed out is that desensitization must be applied under the supervision of an allergologist–immunologist expert and a dedicated nurse [22].

Here, we briefly report the main uses of DD protocols in oncology that we could find through our literature research.

In 2008, Castells et al., reported the safety and efficacy data of a standardized 12-step rapid desensitization protocol developed at their institution, where 413 DD protocols were performed in a case-series of 98 patients with different types of cancer who developed hypersensitive infusion-related reactions to a chemotherapeutic agent or a monoclonal antibody. Patients were desensitized to mainly carboplatin and paclitaxel, but also to liposomal doxorubicin, doxorubicin, cisplatin, oxaliplatin and rituximab. Only 6% of the DD protocols elicited severe reactions, all of which were less severe than the initial reactions and easily managed. All patients succeeded in receiving the full target dose and the protocol was found to be applicable to various types of agent [23]. The same DD protocol was applied in another cohort study focusing on monoclonal antibodies reported by Brennan et al., where 105 DD procedures were administered to 23 patients with oncologic and rheumatologic diseases with hypersensitivity to trastuzumab (3 patients), infliximab (6 patients) and rituximab (14 patients). Only two severe reactions were documented with desensitization, out of thirty overall, and 104 out of 105 drug administrations were therefore completed.

In 2013, another case series was described by Madrigal-Burgaleta et al., where a different rapid DD protocol was applied to oxaliplatin, carboplatin, paclitaxel, docetaxel, cyclophosphamide and rituximab. A total of 188 out of 189 administrations were completed, with no reactions in 94% of cases [24].

Other smaller series, with different DD protocols varying accordingly with the institution considered, have been reported, mainly with respect to classical chemotherapy agents and with consistent results, as reported above.

One crucial question about administering a therapy through a DD protocol in oncology is whether drug efficacy is maintained, considering the considerably different infusion modality of a treatment compared to when it is tested and validated in clinical trials.

Sloane et al. tried to answer these questions by evaluating a large dataset of patients undergoing DD at the Dana Farber Cancer Institute and Brigham and Women’s Hospital from 2007 to 2010, reporting the safety, costs and efficacy of the protocol. With 2177 desensitization protocols performed, involving traditional chemotherapy drugs (platins, paclitaxel, cyclophosphamide) and biological drugs (rituximab, infliximab, trastuzumab, bevacizumab), the safety profiles described in the literature were confirmed and the outcomes maintained, with a nearly 100% completion rate with no or mild reactions in 93% of infusions. The immunology team also managed to desensitize patients starting from a Grade 3 reaction, where permanent treatment discontinuation is usually recommended. With carboplatin being the most used drug, DD efficacy analysis was conducted for this drug, comparing the life-expectancy of 81 carboplatin-desensitized patients who had recurrent ovarian cancer to 155 non-desensitized controls treated with carboplatin for recurrent ovarian cancer as well, using propensity scores. The analysis did not show any significant difference in survival over a range of 5 years after the procedure [25].

In our clinical case, despite skin testing negativity and the impossibility of specifically ruling out the HSR phenotype, we decided to apply DD to maintain the patient’s line of therapy, taking into account the severity of the reaction experienced by the patient and the timing of the onset of the reaction (i.e., at the second infusion), and because DD can also be considered In the case of non IgE-mediated reactions and other HSRs phenotypes, such as cytokine release syndrome [26]. When interpreting our results, it must be considered that skin testing for Cemiplimab is not standardized and that both the sensitivity and specificity of skin testing for this biological agent are unknown. Additionally, we cannot rule out the implication of polysorbate 80, an excipient present in the drug formulation that has been described as causative agent of HSRs in oncology settings [27].

## 4. Conclusions

Our work highlights the need for a desensitization protocol to improve the treatment of infusion-related reactions, particularly when elicited by an immunotherapy agent, prevent treatment discontinuation and maintain treatment efficacy.

## Figures and Tables

**Table 1:** 16-step Cemiplimab desensitization protocol.

Step	Solution (Bag)	Rate of Infusion (mL/h)	Time (min)	Volume Infused (mL)	Dose Administered (mg)/Step	Cumulative Dose (mg)
1	1	2.5	15	0.625	0.000875	0.000875
2	1	5	15	1.25	0.00175	0.002625
3	1	10	15	2.5	0.0035	0.002975
4	1	20	15	5	0.007	0.003675
5	2	2.5	15	0.625	0.00875	0.012425
6	2	5	15	1.25	0.0175	0.029925
7	2	10	15	2.5	0.035	0.064925
8	2	20	15	5	0.07	0.134925
9	3	5	15	1.25	0.175	0.30905
10	3	10	15	2.5	0.35	0.6573
11	3	20	15	5	0.70	1.35695
12	3	40	15	10	1.4	1.75625
13	4	10	15	2.5	3.5	5.2475
14	4	20	15	5	7	12.23
15	4	40	15	10	14	26.195
16	4	80	173	230	324	350
	**Volume (mL)**		**Total Amount of Cemiplimab (mg)**		**Cemiplimab Concentration (mg/mL)**	
Solution 1	250		0.35		0.0014	
Solution 2	250		3.5		0.014	
Solution 3	250		35		0.14	
Solution 4	250		350		1.4	

**Table 2:** Main clinical trials reporting data on HSRs to ICIs in non-melanoma cutaneous cancers.

Author (Ref.)	Year	Design	Type of Cancer	*n*	Intervention	TRAEs (%)	TRAEs ≥ G3 (%)	TR Infusion Reactions (%)	Comments
Midgen [6]	2018	Phase I–II	cSCC	85	Cemiplimab 3 mg/kg every 2 weeks	59 (69)	12 (14.1)	3 (3.5; Grade 1–2)	None
Midgen [7]	2020	Phase II	cSCC	78	Cemiplimab 3 mg/kg every 2 weeks	72 (92.3)	11 (14.1)	NR *	None
Rischin [8]	2020	Phase II	cSCC	56	Cemiplimab 350 mg every 3 weeks	36 (64.3)	7 (12.5)	NR °	None
Grob [12]	2020	Phase II	cSCC	109	Pembrolizumab 200 mg every 3 weeks	70 (66.7)	6 (5.7)	1 (1; Grade 3)	None
Maubec [13]	2020	Phase II	cSCC	55	Pembrolizumab 200 mg every 3 weeks	39 (71)	6 (10.9)	NR	None
Nghiem [14]	2019	Phase II	MCC	50	Pembrolizumab 2 mg/kg every 3 weeks	48 (96)	14 (28)	1 (1)	No specific attribution to treatment.
D’angelo [15]	2018	Phase II	MCC	39	Avelumab 10 mg/kg every 2 weeks	28 (71.8)	8 (20.5)	9 (23.1; 1 Grade ≥ 3)	After specific protocol amendment: premedication with acetaminophen and antihistamine for at least first 4 cycles.
D’angelo [16]	2020	Phase II	MCC	88	Avelumab 10 mg/kg every 2 weeks	68 (77.3)	10 (11.4)	19 (21.6; no Grade ≥ 3)	After specific protocol amendment: premedication with acetaminophen and antihistamine for at least first 4 cycles. Infusion-related reactions led to discontinuation for 2 patients.
Stratigos [17]	2021	Phase II	cBCC	84	Cemiplimab 350 mg every 3 weeks	48 (57)	17 (20)	NR *	None

Abbreviations: ref, reference; TR, treatment-related; TRAEs, treatment-related adverse events; cSCC, cutaneous squamous cell carcinoma; MCC, Merkel cell carcinoma; cBCC, cutaneous basal cell carcinoma; NR, not reported. * at least <10% any grade and none with G ≥ 3. ° at least <5% any grade and none with G ≥ 3.

## Data Availability

Not applicable.
